# High-yield and rapid isolation of extracellular vesicles by flocculation via orbital acoustic trapping: FLOAT

**DOI:** 10.1038/s41378-023-00648-3

**Published:** 2024-02-04

**Authors:** Joseph Rufo, Peiran Zhang, Zeyu Wang, Yuyang Gu, Kaichun Yang, Joseph Rich, Chuyi Chen, Ruoyu Zhong, Ke Jin, Ye He, Jianping Xia, Ke Li, Jiarong Wu, Yingshi Ouyang, Yoel Sadovsky, Luke P. Lee, Tony Jun Huang

**Affiliations:** 1 Department of Mechanical Engineering and Materials Science, Duke University, Durham, NC USA; 2 Department of Biomedical Engineering, Duke University, Durham, NC USA; 3 Department of Obstetrics, Gynecology, and Reproductive Sciences, Magee-Womens Research Institute, University of Pittsburgh, Pittsburgh, PA USA; 4 Department of Microbiology and Molecular Genetics, School of Medicine, University of Pittsburgh, Pittsburgh, PA USA; 5 Renal Division and Division of Engineering in Medicine, Department of Medicine, Brigham and Women’s Hospital, Harvard Medical School, Boston, MA USA; 6 Department of Bioengineering, Department of Electrical Engineering and Computer Science, University of California, Berkeley, Berkeley, CA USA; 7 Department of Biophysics, Institute of Quantum Biophysics, Sungkyunkwan University, Suwon, Korea

**Keywords:** Engineering, Nanoscience and technology

## Abstract

Extracellular vesicles (EVs) have been identified as promising biomarkers for the noninvasive diagnosis of various diseases. However, challenges in separating EVs from soluble proteins have resulted in variable EV recovery rates and low purities. Here, we report a high-yield ( > 90%) and rapid ( < 10 min) EV isolation method called FLocculation via Orbital Acoustic Trapping (FLOAT). The FLOAT approach utilizes an acoustofluidic droplet centrifuge to rotate and controllably heat liquid droplets. By adding a thermoresponsive polymer flocculant, nanoparticles as small as 20 nm can be rapidly and selectively concentrated at the center of the droplet. We demonstrate the ability of FLOAT to separate urinary EVs from the highly abundant Tamm-Horsfall protein, addressing a significant obstacle in the development of EV-based liquid biopsies. Due to its high-yield nature, FLOAT reduces biofluid starting volume requirements by a factor of 100 (from 20 mL to 200 µL), demonstrating its promising potential in point-of-care diagnostics.

## Introduction

Due to the invasive nature of traditional tissue biopsies, as well as their limited ability to detect early-stage tumors, many research efforts over the past decade have focused on the development of liquid biopsies, which are noninvasive, diagnostic tests based on the analysis of circulating factors found in blood, urine, and other biofluids^1^. For example, in the diagnosis of prostate cancer, which is the second most diagnosed cancer among men worldwide^2^, transrectal prostate biopsy remains the standard of care for confirming the presence of a tumor. However, approximately 10% of the transrectal prostate biopsies result in subsequent infections^3^, and high-risk cancers are difficult to accurately identify^4^. Many circulating biomarkers, such as circulating tumor cells, circulating tumor DNA, and extracellular vesicles (EVs), have been identified as promising biomarkers for developing noninvasive liquid biopsies for cancer.

EVs are an emerging class of biomarkers found in high concentrations in urine and blood ( ~ 10^8^ and 10^10^ particles/mL, respectively)^5,6^. EVs contain biologically active molecular cargo: nucleic acids, proteins, lipids, and metabolites; these reflect their cell of origin^7,8^. Urine is a valuable biofluid because, unlike blood, it does not require a needle stick and can be collected in large volumes over multiple longitudinal time points. Recently, the ExoDx Prostate IntelliScore (EPI) test, based on the analysis of extracellular RNA (exRNA) contained in urinary EVs, became the first EV-based liquid biopsy to receive breakthrough device designation from the U.S. FDA^9^. The EPI test is primarily used to risk-stratify patients and can help urologists decide whether to defer or proceed with a tissue biopsy. While this can aid in the reduction of unnecessary procedures for patients deemed to be low risk, high-risk patients still need to undergo a tissue biopsy. Although much progress has been made in developing EV-based liquid biopsies, challenges associated with the effective isolation of intact EVs have prevented them from achieving their full clinical potential, as a replacement of tissue biopsy.

Small extracellular vesicles (sEVs; 40–160 nm) are difficult to isolate with high purity and high yield due to their small size, overlap in physical properties with other nanoscale bioparticles (i.e., low-density lipoproteins), and high phenotypic heterogeneity^10^. Furthermore, the reproducibility and complexity of RNA sequencing are heavily influenced by the EV isolation method used^11^. Based on a survey of recent literature, there are approximately 200 unique EV isolation methods and over 1000 unique protocols to isolate intact EVs from biofluids^12^. Differential ultracentrifugation, size-exclusion chromatography, or a combination of both are the most commonly used methods for isolating EVs from biofluids; however, they require lengthy procedures and specialized equipment and suffer from a trade-off between purity and yield. Alternative approaches, such as polymer precipitation^13^ and immunoaffinity capture^14^, provide simplified procedures and reduced isolation times; nevertheless, polymer precipitation faces challenges with the co-isolation of contaminating proteins^15^, and immunoaffinity capture approaches typically only isolate a subset of EVs^16^, which may bias the downstream analysis. More recently, approaches such as field-flow fractionation^17^ and tangential flow fractionation^18^ have gained popularity due to their ability to isolate highly pure EV subpopulations; however, these approaches require significant sample preprocessing, which typically includes an ultracentrifugation step. No one-size-fits-all approach to the isolation of EVs exists, and protocols need to consider the target biofluids’ unique biochemical and biophysical properties.

The isolation of intact EVs from urine is complicated by the Tamm-Horsfall protein (THP), which is the most abundant urinary protein. THP is a secreted glycoprotein that polymerizes via its zona pellucida domain and forms large filaments up to several microns in length^19^. When isolating urinary EVs via differential ultracentrifugation, the THP polymer network traps EVs during the intermediate ( ~ 20,000 × *g*) centrifugation steps, leading to a loss of approximately 40% of the total EV population^20^. As a result, significantly larger starting volumes ( ~ 10–20 mL) of urine samples from patients are needed. Protocols have been developed to attempt to eliminate the THP contamination and involves using chemical detergents to reduce THP^21^; however, these potent reducing agents can also damage the EV proteins, limiting the use of these protocols in biomarker discovery studies. In this regard, developing a high-purity, high-yield technique for isolating urinary EVs would translate EV biomarkers from research labs into clinical applications.

In this study, we have developed a strategy for the rapid ( < 60 s), high-purity, high-yield ( > 90% recovery rate), selective isolation of EVs from urine samples based on FLocculation via Orbital Acoustic Trapping (FLOAT) to address this need. Flocculation has been extensively used to cluster and separate nanoparticles in applications such as pharmaceutical processing^22^ and mineral recovery^23^. Recently, flocculation has been used to swiftly cluster EVs prior to filtration, providing a rapid approach for EV isolation^24^. However, this approach requires mechanical filtration and elution. To date, flocculation has yet to be implemented for the direct isolation of EVs. Our FLOAT approach relies on a thermoresponsive polymer, poly(*N*-isopropylacrylamide) (PNIPAm). Notably, this polymer’s amine-terminated, cationic form was selected to more efficiently adsorb to the surface of the negatively charged EVs^25^. The gold-standard method in the field of ultracentrifugation requires starting volumes of 100 mL; however, our FLOAT platform is capable of isolating sEVs from as low as 10 µL of urine (1000× reduction). By significantly reducing the starting sample volume and minimizing protein contamination, we have addressed two fundamental difficulties that have, thus far, hindered the use of EVs in fundamental and clinical research settings. Our FLOAT platform does not only simplify the isolation of EVs, but we anticipate FLOAT will have broader scientific applications in the fields of biology, chemistry, physics, engineering, materials science, and medicine.

## Results

### Physical mechanism of FLOAT

**Fig. 1:**
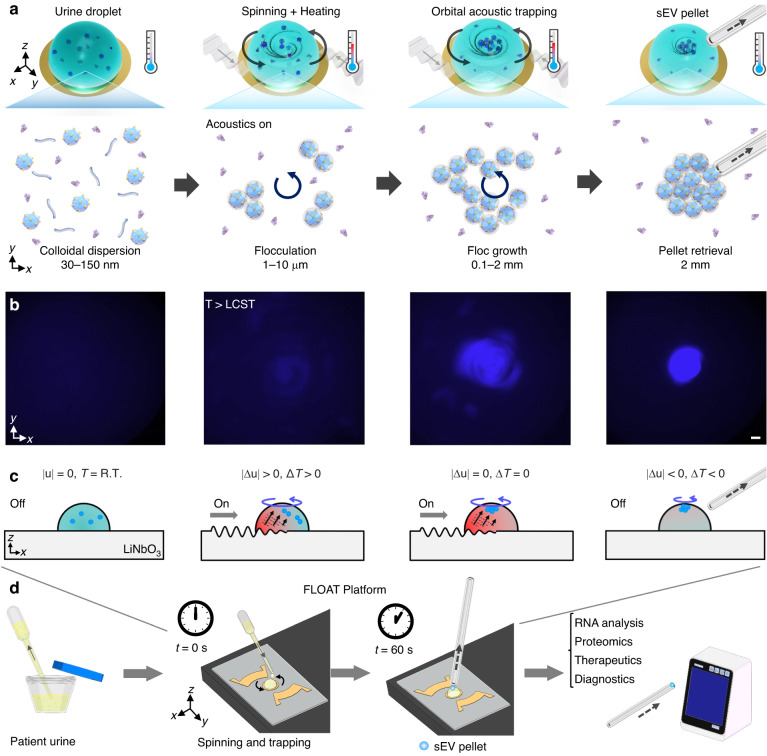
Mechanism of FLocculation via Orbital Acoustic Trapping (FLOAT) method for the rapid isolation and efficient concentration of EVs. **a** Schematic depicting the heating and rotation process. Initially, small extracellular vesicles (sEVs) are randomly distributed throughout the droplet. When the acoustic transducers are turned on, the droplet rotates and heats. Once the droplet temperature rises above the lower critical solution temperature of PNIPAm (~32 °C), the flocculation process begins. As the droplet continues to rotate, particle flocs collide and merge, eventually forming a single particle floc at the center of the droplet. This floc can be manually transferred and resuspended in another buffer for subsequent analysis. (sEVs: small extracellular vesicles; *T*: droplet temperature; LCST: lower critical solution temperature). **b** Microscope images showing the concentration of the fluorescently labeled urinary sEVs inside a rotating liquid droplet. Scale bar: 200 µm. **c** Schematic depicting the various stages of the FLOAT process. **d** Sample to analysis pipeline for the concentration and isolation of urinary EVs

A schematic of the FLOAT process is shown in Fig. 1a. The underlying process of flocculation^26–28^ relies on the adsorption of a polymer to the surface of a solid suspended in the colloid form. Due to an external trigger, such as a change in heat or pH, the flocculant becomes less soluble. It precipitates out of the solution^29^, enabling the collection of the suspended solids. To achieve EV isolation, the PNIPAm polymer solution (1% w/v in deionized water) was mixed in equal parts with a cell-free urine sample. A microliter-sized droplet of this mixed solution was added to our acoustofluidic device^30–50^. Acoustofluidic devices have previously been developed to isolate EVs from whole blood^42^ and saliva^43^ in a contact-free manner. These microfluidic devices sequentially filter biofluid samples and remove particles larger than EVs; however, the isolated samples contain soluble proteins, which may interfere with downstream analyses. Furthermore, these devices utilize syringe pumps and high operating voltages (45 *Vpp*) and, as a result, require external cooling to prevent damage to the samples. These requirements limit the portability of such devices and their potential use in point-of-care applications.

In our design, two pairs of focused acoustic transducers with laterally offset focal points are positioned on either side of the liquid droplet. When the device is turned on, the acoustic waves propagate toward the liquid droplet and couple into the fluid, enabling controllable heating and rotation of the droplet. Once the droplet temperature is raised above the lower critical solution temperature (LCST) for PNIPAm of ~32 °C, the polymer undergoes a reversible coil-to-globule phase transition^51^. As a result of this conformational change, the polymer experiences a change in hydrophobicity from hydrophilic to hydrophobic; this results in the generation of attractive hydrophobic forces between the coated particles. Because the droplet is also rotating, polymer-coated EVs continuously collide with each another and aggregate, forming particle flocs. These flocs ultimately aggregate into a single pellet that becomes trapped at the droplet’s center; this pellet can be manually removed with a glass microcapillary and resuspended in phosphate-buffered saline (PBS). This acoustofluidic approach enables a simple, fast, and efficient strategy for isolating EVs from complex biofluids with low levels of protein contamination (Fig. 1b). Different from ultracentrifugation approaches, which result in the trapping of EVs in the THP polymer network, FLOAT can directly isolate urinary EVs from THP. Due to the small size and portable nature of our acoustofluidic centrifuge (Fig. 1d), FLOAT is a highly accessible, user-friendly technology that can expand the use of EVs in biomedical research.

### Experimental verification of FLOAT

**Fig. 2:**
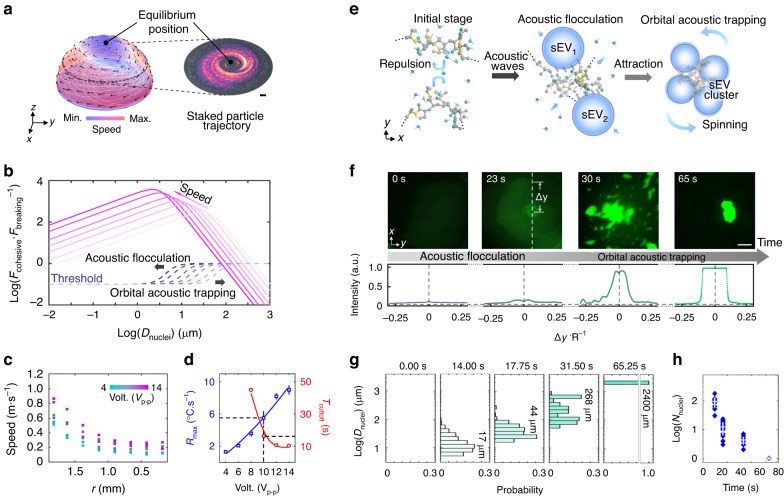
Characterization of the FLOAT method. **a** Simulation results and stacked particle images of the trajectories of particles within a rotating droplet. Particles are concentrated at an equilibrium position located at the top and center of the droplet. **b** Theoretical analysis of the various cohesive and breaking forces that govern the flocculation process. A detailed discussion is provided in Supplementary Note S1. **c** Rotational speed and temperature as a function of applied voltage. **d** Heating rate and cutoff temperature as a function of input voltage. **e** Molecular mechanism of PNIPAm-based flocculation. Initially, when the droplet temperature is below 32 °C, the PNIPAm polymer is in a hydrophilic configuration. Repulsive forces between PNIPAm molecules adsorbed onto the surface of sEVs prevent their aggregation. However, once acoustic waves are applied and the temperature is raised above 32 °C, the PNIPAm polymer undergoes a coil-to-globule transition, resulting in a hydrophobic state. The hydrophobic attraction between molecules enables the flocculation of the sEV particles and the formation of the sEV clusters. **f** Time-lapse images of flocculation *via* orbital acoustic trapping of 50 nm fluorescent polystyrene particles. Scale bar: 200 µm. **g** Analysis of the size and number of the particle clusters over time from the 50 nm polystyrene particle experiment. **h** Number of the particle flocs as a function of time from the 50 nm polystyrene particle experiment

We conducted COMSOL Multiphysics® simulations to predict the acoustic streaming and the trajectory of the nanoparticles within the rotating droplet (Fig. 2a; left). Compared with stacked particle trajectories obtained from the microscopy images (Fig. 2a; right), the predicted particle trajectories agreed well with the experimental observations. We also modeled the cohesive and breaking forces that determine the equilibrium diameter of the particle floc and investigated the effect of droplet rotation speed on the equilibrium diameter (Fig. 2b, Supplementary Note S1). As the droplet rotation speed increased, the cohesive forces increased, resulting in a more tightly bound, smaller particle floc. We experimentally characterized the droplet rotation speed and temperature as a function of input voltage (Fig. 2c, d). Because the LCST of the PNIPAm flocculant was ~32 °C, we selected 10 *Vpp* as our operating voltage when processing urine samples. Under these conditions, the droplet reached the LCST in ~ 30 s, and the temperature of the droplet stabilized at ~37 °C, around the physiological temperature of the urine. Figure 2e shows the reversible behavior of the adsorbed PNIPAm polymer on the EV surface when it is above and below the LCST. Initially, when the temperature is below the LCST, the cationic polymer adsorbs to the surface of the negatively charged EVs and acts as a steric stabilizer. When the acoustics are turned on and the droplet is heated, the PNIPAm undergoes a coil-to-globule transition, enabling the flocculation of the surrounding particles. This coil-to-globule transition is accompanied by an increase in the zeta potential, as shown in Supplementary Fig. S2, demonstrating the ability of PNIPAm to neutralize the charge of typically negatively charged EVs.

Figure 2f shows time-lapse images of the flocculation process for the 50 nm fluorescent polystyrene beads mixed with the PNIPAm solution. As the temperature of the droplet crossed the LCST, fluorescent flocs became visible in the fluid. By 65 s, most of the 50 nm particles were concentrated at the center of the droplet (Supplementary Fig. S3, Supplementary Tables 1 and 2). At these short time scales, no noticeable evaporation of the droplet occurred throughout the FLOAT process. This process was further analyzed to assess the number of flocs throughout the FLOAT process. As shown in Fig. 2g, h, the flocs did not appear until the temperature crossed the LCST and demonstrated that all the flocs were concentrated into a large polymer aggregate at the center of the droplet. To confirm that the concentration process was a result of FLOAT and not purely acoustic concentration, we conducted identical experiments with particles in water, and the acoustofluidic centrifuge alone was unable to concentrate particles (Supplementary Fig. S4).

### Isolating urinary EVs via FLOAT

**Fig. 3:**
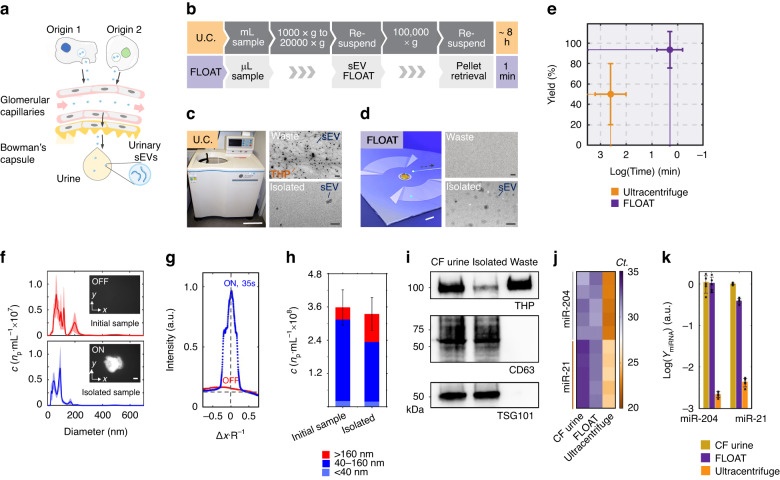
Biological characterization of the FLOAT platform. **a** Schematic showing how various cell types secrete small extracellular vesicles (sEVs) into urinary circulation, enabling the detection of biomarkers for multiple disease states. **b** Comparison between ultracentrifugation (U.C) and FLOAT protocols for isolating sEVs from cell-free urine. Comparison between the instruments needed for **c** ultracentrifugation and **d** FLOAT and the TEM samples obtained from each approach. In the waste portion from the samples isolated via ultracentrifugation, many sEVs are trapped inside the THP networks, resulting in low yield. On the other hand, sEVs are not observed in the waste portion of the samples isolated via FLOAT due to the high capture efficiency of flocculation. Scale bars: **c**, **d** TEM: 200 nm; **c** picture: 300 mm; **d** picture: 3 mm. **e** Comparison between ultracentrifugation and FLOAT in terms of yield and processing time. **f** NTA results showing the size distribution of particles in the original urinary sEV sample and isolated urinary sEV sample. Insets show fluorescence images of the sEVs before and after applying acoustic waves. Scale bar: 200 µm. **g** Fluorescence intensity of the fluorescently labeled EVs before and after FLOAT concentration. After acoustic waves are applied, the EVs form a highly concentrated polymer floc at the center of the droplet. **h** Concentration and size distribution of the particles in the initial cell-free urine samples and FLOAT isolated samples. Each set of the NTA data was obtained from at least three NTA assays. Ten subsamples are measured and combined for each sample used for NTA measurement to provide statistically relevant data (see Figure S6 for alternatively displayed NTA data). **i** Western blot results showing the EV markers (CD63 and TSG101) in the FLOAT isolated samples along with the absence of THP. **j** miRNA analysis comparing ultracentrifugation with FLOAT. **k** Average Ct values for FLOAT compared with those obtained using ultracentrifugation, indicating the high-yield nature of FLOAT

Urinary EVs contain biomarkers for various diseases, including prostate cancer^52^, kidney diseases^53^, bladder cancer^54^, and neurodegenerative diseases^55^. These EVs can be released from various cell types (Fig. 3a) and are excreted in the urine. A comparison between the traditional ultracentrifugation protocols and our FLOAT protocol is shown in Fig. 3b. While standard protocols take at least 8 hours to isolate samples, our FLOAT approach can isolate EVs in as little as 1 minute. We compared conventional ultracentrifugation with our acoustic centrifuge, as shown in Fig. 3c, d. The smaller footprint of our acoustic centrifuge device enables it to be used in portable applications. Notably, we compared the transmission electron microscopy (TEM) images of samples isolated from each method. In the waste from differential centrifugation (i.e., 20,000 × *g* pellet), the EVs are trapped in the THP network and would typically be discarded (Supplementary Fig. S4). The EVs could not be located on the TEM grids of the waste from the FLOAT method. Furthermore, the EVs isolated via ultracentrifugation are scarce compared to the fraction isolated via FLOAT. A comparison between the yield (93% vs. 50%) and processing time (1 min vs. 480 min) of FLOAT and ultracentrifugation is shown in Fig. 3e. Notably, the yield calculations are based on particle concentration rather than the total number of particles. As a result, for applications requiring the processing of large volumes or a high number of total particles, ultracentrifugation may be advantageous; however, FLOAT is uniquely well suited for the rapid, high-yield isolation of EVs from low-volume samples, meeting the sample processing needs for many point-of-care diagnostic applications.

To visualize the FLOAT process, we concentrated and isolated the fluorescently labeled urinary EVs (Fig. 3f inset) to evaluate the concentration performance of the polymer system and analyzed the size distribution and yield of the isolated EV fractions. From Fig. 3g, the FLOAT platform could enhance the fluorescence intensity by a factor of ~80 after only 35 s of applying acoustic waves. Nanoparticle tracking analysis (NTA) confirmed the yield and size distribution of EVs in the isolated samples (Fig. 3f, Supplementary Table 3). To assess the yield of the FLOAT method, we isolated sEVs from the cell-free urine samples (Supplementary Fig. S5). Mean particle counts between the initial cell-free urine sample (3.58 × 10^8^ particles/mL) and the FLOAT isolated sample (3.35 × 10^8^ particles/mL) are shown in Fig. 3h. Western blot analysis was used to confirm that the isolated samples contained the EV markers CD-63 and TSG101 and had only small amounts of contaminating THP protein (Fig. 3i). Finally, we conducted reverse transcription quantitative polymerase chain reaction (RT‒qPCR) to evaluate the ability of our OAC platform for the isolation of the microRNA (miRNA) targets. In this study, two generic miRNA targets, miR-21 and miR-204, were used, and these have been implicated as biomarkers for various cancers^56^. These results are shown in Fig. 3j, k. For both targets, our platform could isolate not only miRNA biomarkers but also these targets with a significantly higher yield than ultracentrifugation. When using FLOAT, the average cycle thresholds (Ct) for miR-21 and miR-204 were 23.1 ± 0.2 and 23.6 ± 0.6, respectively. When using ultracentrifugation, the average cycle thresholds (Ct) for miR-21 and miR-204 were 29.7 ± 0.4 and 32.4 ± 0.2, respectively. The lower C_t_ values for FLOAT indicate more starting material in the initial samples, providing a qualitative comparison between the yields of both techniques. A C_t_ value of 35 is typically used as the cutoff for the detection level of RT‒qPCR assays^57^. The low C_t_ values achieved by our FLOAT platform indicated that it could isolate enough EVs for downstream biological analysis, which could be particularly useful for practical diagnostic applications requiring the detection and profiling of urinary EV miRNAs.

## Discussion

By combining acoustofluidics and thermoresponsive nanomaterials, we have developed a simple, rapid, high-yield approach for isolating urinary EVs. Importantly, our FLOAT approach can separate EVs from the most abundant urinary protein, THP; this is an accomplishment that ultracentrifugation cannot reliably achieve. Furthermore, because it does not require multiple low-speed centrifugation steps, the yield of EVs exceeds 90%, enabling the identification of EV biomarkers from as little as 200 µL of fluid. We have demonstrated the ability of our FLOAT platform to concentrate nanoparticles at concentrations ranging between 10^7^ and 10^9^ particles/mL (Supplementary Fig. S8); note that the typical concentration of urinary EVs in patient samples ranges between 10^8^ and 10^9^ particles/mL^58^. Through extensive characterization, we have demonstrated the highly repeatable and robust nature of our FLOAT platform. Future work will optimize our FLOAT platform for use with other biofluids, such as plasma and saliva, and the impact of factors such as polymer concentration, salt concentration, and sample pH on EV flocculation will be investigated. Notably, although our FLOAT platform is capable of selectively separating urinary EVs from proteins such as THP and albumin (Supplementary Fig. S9a), it is less selective when separating EVs from lipoproteins (Supplementary Fig. S9b). Fortunately, the concentration of lipoproteins found in most urine samples is relatively low (0.1–1 ng/mL) when compared to the concentrations found in plasma samples (0.1–1 mg/mL)^59^. As a result, the low amounts of lipoproteins in the isolated urinary EV samples did not interfere with downstream analysis. However, for our FLOAT platform to use biofluids such as plasma, we must improve the selectivity toward EVs by exploiting minute differences in the zeta potential and size. Additional areas for exploration include adding a wash step to remove excess PNIPAm polymer and miRNAs that may adsorb to the surface during the isolation process and continuous flow configurations to enable the processing of larger volume samples. An approach that has shown promise in eliminating cationic polymers from EVs is using elution buffers containing chaotropic agents, such as guanidium thiocyanate, and anionic membranes to collect the washed polymers^24^. Furthermore, we can incorporate disposable thin-film polydimethylsiloxane (PDMS) superstrates that can be placed on top of the piezoelectric substrate^60,61^ to enable the FLOAT to become reusable; this will eliminate the need for manual cleaning between each sample. In applications that require large volume samples, we envision that the high-yield and rapid nature of FLOAT will make it a suitable candidate to be combined with other isolation protocols (such as those incorporated downstream of ultracentrifugation) to remove contaminating proteins and facilitate high-purity EV sample preparation.

Overall, by significantly reducing the starting sample volume by a factor of 100 (20 mL to 200 µL) and eliminating the issue of THP trapping, we have addressed the two fundamental difficulties that have, thus far, hindered the development of urinary EV-based liquid biopsies. Furthermore, the FLOAT platform is highly portable and can be operated using only a function generator and amplifier (pumps or other microfluidic accessories are not needed), enabling its use in remote and clinical settings. Alternative approaches, such as ultracentrifugation, require specialized laboratory equipment, which limits their use in point-of-care applications and remote settings. We anticipate that FLOAT has tremendous potential in expediting the development of EV-based molecular diagnostics and can help to achieve the potential of EVs as a point-of-care biomarker.

## Materials and methods

### Device design and fabrication

The focused interdigital transducers (IDTs; 5 nm Cr and 50 nm Au) were fabricated on a 128° Y-cut lithium niobite (LiNbO_3_) wafer (Precision Micro-Optics, USA) using standard photolithography followed by electron beam evaporation and a lift-off process. The concentric electrode finger width and spacing were kept constant at 50 µm, resulting in an operating frequency of ~20 MHz. Each transducer contained 40 pairs of interdigitated electrodes. A ring (4 mm inner diameter, 5 mm outer diameter, 5 nm Cr, and 50 nm Au) was deposited at the center of the IDTs to denote the droplet holding area. The focal point of each pair of IDTs was offset 1.5 mm from the center of the ring, achieving a lateral separation of 3 mm. When a liquid droplet (volume: 5–15 µL) was placed in the droplet holding area and the acoustic transducers were turned on, the droplet rotated around its central axis. Due to the equally offset focal points of the transducers, the droplet tended to self-focus at the center of the droplet holding area. The IDTs were connected to wires using silver epoxy (MG Chemicals, USA). A function generator (DG 3012 C, Teletronics Technology Corporation, USA) and amplifier (25A250A, Amplifier Research, USA) were used to activate both pairs of focused IDTs and to generate surface acoustic waves.

### Small extracellular vesicle isolation protocol via FLOAT

Pooled urine samples (Lee Biosolutions, USA) were thawed on ice and processed as follows:

1. Urine was thawed in a 37 °C water bath until all the ice crystals in the tubes disappeared. After thawing was complete, the samples were mixed by gentle vortexing for 10 s.
2. The whole urine sample was centrifuged at 2000 × *g* for 10 min at 37 °C.
3. The supernatant was collected, and the pellet was discarded. The collected supernatant is referred to as the cell-free urine sample.
4. Cell-free urine samples were combined with an equal volume of FLOAT isolation buffer (1% w/v amine-terminated PNIPAm (Millipore Sigma, USA) in deionized water). Note: if only EVs smaller than 200 nm (i.e., sEVs) needed to be collected, the combined sample and isolation buffer were filtered through a 0.22 µm syringe filter (Millipore Sigma, USA).
5. Droplets ranging in volume from 5 to 10 µL were applied to the acoustic centrifuge device.
6. Acoustic power was applied at 10 V_pp_.
7. After the droplet rotated for ~60 s and the pellet was visually observed, a glass capillary (VitroCom, USA) was used to retrieve the pellet.
8. The EV pellet was resuspended in a suitable resuspension buffer, such as 1×phosphate-buffered saline (PBS), and analyzed using NTA, TEM, and Western blotting.
9. Steps 5–8 were repeated until the needed total volume of sample was processed. Note: because the sample was diluted 1:1 with FLOAT isolation buffer, the sample volume processed was half of the total droplet volume processed.
10. The FLOAT device was cleaned with 70% isopropanol and rinsed with deionized water.

### Small extracellular vesicle isolation protocol via ultracentrifugation

Pooled urine samples (Lee Biosolutions, USA) were processed following established protocols ^19,20^.

The protocol for obtaining sEVs isolated by ultracentrifugation is as follows:

1. Urine was thawed in a 37 °C water bath until all the ice crystals in the tubes disappeared. After thawing was complete, the samples were mixed by gentle vortexing for 10 s.
2. Fifty milliliters of urine was centrifuged at 2000 × *g* for 10 min at 37 °C.
3. The supernatant was collected, and the pellet was discarded. The collected supernatant is referred to as the cell-free urine sample.
4. The supernatant was centrifuged at 20,000 × *g* for 30 min at 37 °C. The supernatant was collected, and the pellet was discarded.
5. The supernatant was centrifuged at 200,000 × *g* for 2 h at 37 °C. The supernatant was discarded. The pellets were resuspended in 0.5 mL of PBS per tube and analyzed using NTA, TEM, and Western blotting.

### Theoretical and numerical models of internal droplet streaming

The acoustic streaming in the droplet is governed^62,63^ by the continuity equation and Navier‒Stokes equation:

$${\rho }_{0}\nabla \cdot {\boldsymbol{v}}=0$$

$${\rho }_{0}\left({\boldsymbol{v}}\cdot \nabla \right){\boldsymbol{v}}=-\nabla p+\mu {\nabla }^{2}{\boldsymbol{v}}+\left({\mu }_{b}+\frac{1}{3}\mu \right)\nabla \left(\nabla \cdot {\boldsymbol{v}}\right)+{\boldsymbol{F}}$$

where ${\boldsymbol{v}}$ and $p$ are the velocity of the stream and the pressure in the droplet, respectively, and ${\rho }_{0}$, *μ*, and *μ*_*b*_ are the fluid density, dynamic viscosity, and bulk viscosity, respectively. An acoustic wave-activated body force (${\boldsymbol{F}}$) is applied as the driving force to the acoustic streaming. The body force^62,63^ can be expressed by the following:

$${F}_{x}=-{\rho }_{0}\left(\frac{\partial \bar{{v}_{x1}{v}_{x1}}}{\partial x}+\frac{\partial \bar{{v}_{y1}{v}_{x1}}}{\partial y}+\frac{\partial \bar{{v}_{z1}{v}_{x1}}}{\partial z}\right)$$

$${F}_{y}=-{\rho }_{0}\left(\frac{\partial \bar{{v}_{x1}{v}_{y1}}}{\partial x}+\frac{\partial \bar{{v}_{y1}{v}_{y1}}}{\partial y}+\frac{\partial \bar{{v}_{z1}{v}_{y1}}}{\partial z}\right)$$

$$\,{F}_{z}=-{\rho }_{0}\left(\frac{\partial \bar{{v}_{x1}{v}_{z1}}}{\partial x}+\frac{\partial \bar{{v}_{y1}{v}_{z1}}}{\partial y}+\frac{\partial \bar{{v}_{z1}{v}_{z1}}}{\partial z}\right)$$

where ${v}_{x1}$, ${v}_{y1}$, and ${v}_{z1}$ are the x, y, and z components of ${{\boldsymbol{v}}}_{{\bf{1}}}$, which is the acoustic particle velocity expressed by the following:

$${v}_{x1}=0$$

$${v}_{y1}=i\omega \left({A}_{m}{e}^{i\omega t}{e}^{-i{k}_{L}y}{e}^{-\alpha {k}_{L}z}\right)$$

$${v}_{z1}=i\omega \left({-i\alpha A}_{m}{e}^{i\omega t}{e}^{-i{k}_{L}y}{e}^{-\alpha {k}_{L}z}\right)$$

where ${A}_{m}$ is the amplitude of substrate vibration, ${k}_{L}$ is the wavenumber of the acoustic waves, and $\alpha$ is the attenuation coefficient of acoustic waves. By substituting the expressions of the acoustic particle velocity, the acoustic wave-activated body force (${\boldsymbol{F}}$) can be further expressed by the following:

$${F}_{x}=0$$

$${F}_{y}=-\left(1+{\alpha }_{1}^{2}\right){A}_{m}^{2}{\omega }^{2}{k}_{i}{e}^{[2\left({k}_{i}y+{\alpha }_{1}{k}_{i}z\right)]}$$

$${F}_{z}=-\left(1+{\alpha }_{1}^{2}\right){A}_{m}^{2}{\omega }^{2}{k}_{i}{\alpha }_{1}{e}^{[2\left({k}_{i}y+{\alpha }_{1}{k}_{i}z\right)]}$$

where $\alpha =i{\alpha }_{1}$. Note that our simulation uses different coordinates than Figs. 1, 2. Specifically, the surface acoustic waves propagate on the surface of the substrate along the y-axis, and the x-axis and z-axis are perpendicular to the wave propagation direction and the substrate surface, respectively.

The “Laminar Flow” module in the Finite Element Method-based software package COMSOL Multiphysics 5.4 (COMSOL AB, Sweden) was utilized to solve the governing equations of acoustic streaming in the droplet. The expression for the body force was applied to the fluid field by adding a “volume force” condition. The boundary condition at the bottom of the droplet was set to “no slip,” which indicates ${\boldsymbol{v}}={\bf{0}}$, and the boundary condition at the dome of the droplet was set to “slip,” which indicates ${\boldsymbol{v}}\cdot {\boldsymbol{n}}=0$. A “stationary” solver was applied to solve the numerical model.

### Image acquisition and analysis

Microscopic images and videos were acquired using an upright BX51WI microscope (Olympus, Japan) combined with a CoolSNAP HQ2CCD camera (Photometrics, USA). The high-speed videos needed to capture the droplet rotation speed (Fig. 2), and an inverted microscope (TE2000-U, Nikon, Japan) equipped with a fast camera (Photron, Japan) was used. The droplet spinning motion was captured with a frame rate of 3000 fps and analyzed using ImageJ (National Institutes of Health (NIH), USA) and MATLAB R2016b (MathWorks, USA). The sEV samples were collected and visualized using TEM (FEI Tecnai G2 Twin, FEI Company, USA) with a negative staining method. The nanoparticle size distribution and concentration pre- and postprocessing were analyzed using a Malvern Zetasizer (Malvern Instruments, UK) and NTA with a NanoSight LM10 apparatus (Amesbury, UK) following the manufacturers’ protocols. For Zetasizer analysis, the samples were diluted 40× prior to analysis. For NanoSight analysis, 300 µL of the undiluted samples were directly loaded into the sample chamber using a 1 mL syringe. The camera level was set to 16, and the slider shutter was set to 1300. The NanoSight LM10 recorded 10- and 60-second videos for each sample, which were then analyzed using nanoparticle tracking analysis (NTA) 2.0 Analytical software. For sample analysis, the detection threshold was set to level 3.

### Droplet generation and sample preparation

Liquid droplets were deposited onto the acoustofluidic centrifuge using adjustable volume pipettes (Fisher Scientific, USA). For the nanoparticle concentration experiments, fluorescent polystyrene particles with diameters of 100-nm-, 51-nm-, and 28-nm-diameter PS particles (Sigma‒Aldrich, USA; Bangs Laboratories, USA) and different fluorescence tags were used. To visualize the sEV concentration and isolation process, fluorescently labeled urinary exosomes (BioVision, USA) were used.

### Temperature measurements

We used a handheld digital thermometer (Omega, USA) to measure the temperature of the liquid droplets. The thermocouple was suspended above the substrate, and the tip of the thermocouple was immersed in the liquid droplet. This setup eliminated possible unwanted electrical interference from the LiNbO_3_ substrate. The baseline temperature was stabilized for 60 s before each measurement.

### Western blot analysis

sEV samples isolated via FLOAT and sEV samples isolated via ultracentrifugation from equal volumes of cell-free urine were analyzed for sEV markers and circulating proteins. Twenty microliters of each sample was lysed in Pierce Cell Lysis Buffer (Thermo Fisher Scientific, USA) with Halt Protease Inhibitor Cocktail (Thermo Fisher Scientific, USA). Lysates were processed by SDS/PAGE and transferred to a polyvinylidene fluoride membrane (Bio-Rad, USA). Primary antibodies, including mouse anti-CD63 (Santa Cruz Biotechnology, USA), mouse anti-THP (Santa Cruz Biotechnology, USA), and rabbit anti-TSG101 (Abcam, USA), were separately used to incubate the membrane for 12 h at 4 °C. Appropriate horseradish peroxidase secondary antibodies, including goat anti-mouse IgG and goat anti-rabbit IgG (Abcam, USA), were used for a 1-h incubation at room temperature. ChemiDoc XRS+ (Bio-Rad, USA) was used to characterize protein expression levels.

### Enzyme-linked immunosorbent assay (ELISA)

We quantified the concentrations of albumin and lipoprotein (a) using enzyme-linked immunosorbent assay (ELISA) kits (ab227933 and ab212165, Abcam, USA) according to the manufacturer’s instructions. Briefly, serially diluted standards were prepared with two replicates. Then, 50 µL of standards and samples were added to the appropriate wells, and 50 µL of the antibody cocktail was added to each well. Following 1 h of incubation, each well was washed three times. Next, 100 µL of the TMB development solution was added to each well and incubated in the dark for 10 min. After adding 100 µL of the stop solution, the OD value of each well was recorded using a spectrophotometric microplate reader (BioTek Instruments, Inc.) at a wavelength of 450 nm. The final concentration from each sample was calculated using the standard curve. In each case, we quantified the concentration of albumin and lipoprotein in the original cell-free urine samples, the isolated EV samples, and the waste solution.

### Nucleic acid extraction and real-time PCR

Real-time PCR was performed on the 7900HT Fast Real-Time PCR System (Thermo Fisher Scientific, USA). According to the manufacturer’s instructions, exosomal RNA was extracted using the miRNeasy Micro Kit (QIAGEN, Germany). Small RNA TaqMan™ assays (Thermo Fisher Scientific, USA) for miR-21 and miR-204 were used as primers. Reverse transcription was conducted with a TaqMan™ MicroRNA Reverse Transcription Kit (Thermo Fisher Scientific, USA). Reagents and samples were mixed according to the manufacturer’s instructions and aliquoted into a 96-well plate (Applied BioSystems, USA); the well plate was then sealed with an optical adhesive cover (Applied BioSystems, USA). Three replicate reactions per target per sample were used. Default thermal cycling settings were used (initial activation: 2 min at 50 °C followed by 10 min at 95 °C; 40 PCR cycles: 15 s at 95 °C to melt followed by 1 min at 60 °C to anneal). Sequence Detection Systems (SDS) Automation Controller Software v2.3 was used to analyze fluorescence intensity data and obtain the corresponding C_T_ values.

### Calculation of the particle recovery rate and particle shell thickness

The particle recovery, *R*, rate is defined as the concentration of the particles in the FLOAT isolated sample, *N*_*FLOAT*_, divided by the concentration of particles in the initial sample, *N*_*i*_ (Supplementary Table 2).

1 $$R=\frac{{N}_{{FLOAT}}}{{N}_{i}}$$

The thickness, *t*, of the PNIPAm shell can be obtained from the change in the average radius between an unprocessed sample, *r*_*i*,_ and the average radius of the FLOAT isolated sample, *r*_*FLOAT*_, is measured by DLS (Supplementary Table 2). Identical laser illumination conditions are used for each sample to ensure equal interrogation conditions. The shell thickness is estimated by the following:

2 $$t={r}_{{FLOAT}}-{r}_{i}$$

### Supplementary information

Supplementary Information

Supplementary movie S1

Supplementary movie S2

Supplementary movie S3

## Data Availability

All the data supporting the findings of this study are available in the article and its Supplementary Information. All experiments were performed at least in triplicate. Data are shown as the mean ± SD. Further information is available from the corresponding author upon request.

## Code availability

The acoustic wave simulations were performed with the commercial software COMSOL Multiphysics®. Data analysis was performed using MATLAB. The parameters used in this analysis can be found in the Supplementary Information. Computational details can be made available from the corresponding authors upon request.

## References

[1] Bardelli A, Pantel K (2017). Liquid biopsies, what we do not know (yet). Cancer Cell.

[2] Wang L (2022). Prostate cancer incidence and mortality: global status and temporal trends in 89 countries from 2000 to 2019. Front. Public Health.

[3] Johansen TEB (2020). Antibiotic resistance, hospitalizations, and mortality related to prostate biopsy: first report from the Norwegian Patient Registry. World J. Urol..

[4] D’Elia C (2014). Upgrading and upstaging in prostate cancer: from prostate biopsy to radical prostatectomy. Mol. Clin. Oncol..

[5] Musante L (2020). Rigorous characterization of urinary extracellular vesicles (uEVs) in the low centrifugation pellet-a neglected source for uEVs. Sci. Rep..

[6] Johnsen KB, Gudbergsson JM, Andresen TL, Simonsen JB (2019). What is the blood concentration of extracellular vesicles? Implications for the use of extracellular vesicles as blood-borne biomarkers of cancer. Biochim. Biophys. Acta..

[7] Van Niel G, d’Angelo G, Raposo G (2018). Shedding light on the cell biology of extracellular vesicles. Nat. Rev. Mol. Cell Biol..

[8] A. C. Dixson, T. R. Dawson, D. Di Vizio, A. M. Weaver, Context-specific regulation of extracellular vesicle biogenesis and cargo selection. *Nat. Rev. Mol. Cell Biol.* 1–23 (2023). 10.1038/s41580-023-00576-0 PMC10330318 36765164

[9] C. Happel, A. Ganguly, D. A. Tagle, Extracellular RNAs as potential biomarkers for cancer. *J. Cancer Metastasis Treatment* **6**, (2020). 10.20517/2394-4722.2020.71 PMC7821910 33490601

[10] Wang J (2021). Characterizing the heterogeneity of small extracellular vesicle populations in multiple cancer types via an ultrasensitive chip. ACS Sens..

[11] Srinivasan S (2019). Small RNA sequencing across diverse biofluids identifies optimal methods for exRNA isolation. Cell.

[12] Van Deun J (2017). EV-TRACK: transparent reporting and centralizing knowledge in extracellular vesicle research. Nat. Methods.

[13] Garcia-Romero N (2019). Polyethylene glycol improves current methods for circulating extracellular vesicle-derived DNA isolation. J. Transl. Med..

[14] Liu C, Su C (2019). Design strategies and application progress of therapeutic exosomes. Theranostics.

[15] Kalra H (2013). Comparative proteomics evaluation of plasma exosome isolation techniques and assessment of the stability of exosomes in normal human blood plasma. Proteomics.

[16] Rupp A-K (2011). Loss of EpCAM expression in breast cancer derived serum exosomes: role of proteolytic cleavage. Gynecologic Oncol..

[17] Zhang H, Lyden D (2019). Asymmetric-flow field-flow fractionation technology for exomere and small extracellular vesicle separation and characterization. Nat. Protoc..

[18] Busatto S (2018). Tangential flow filtration for highly efficient concentration of extracellular vesicles from large volumes of fluid. Cells.

[19] Fernández-Llama P (2010). Tamm-Horsfall protein and urinary exosome isolation. Kidney Int..

[20] Xu X (2019). Management of Tamm–Horsfall protein for reliable urinary analytics. PROTEOMICS–Clin. Appl..

[21] Musante L (2012). Biochemical and physical characterisation of urinary nanovesicles following CHAPS treatment. PLoS ONE.

[22] Burgstaller D (2018). Continuous cell flocculation for recombinant antibody harvesting. J. Chem. Technol. Biotechnol..

[23] Ng WS, Sonsie R, Forbes E, Franks GV (2015). Flocculation/flotation of hematite fines with anionic temperature-responsive polymer acting as a selective flocculant and collector. Miner. Eng..

[24] Kim J, Lee H, Park K, Shin S (2020). Rapid and efficient isolation of exosomes by clustering and scattering. J. Clin. Med..

[25] Liu R, Saunders B (2009). Thermoresponsive surfaces prepared using adsorption of a cationic graft copolymer: a versatile method for triggered particle capture. J. Colloid Interface Sci..

[26] Gregory J, O’Melia CR (1989). Fundamentals of flocculation. Crit. Rev. Environ. Sci. Technol..

[27] Thomas D, Judd S, Fawcett N (1999). Flocculation modelling: a review. Water Res..

[28] Bratby, J. Coagulation and flocculation. *Uplands: Croydon, England*, (1980).

[29] Plunkett KN, Zhu X, Moore JS, Leckband DE (2006). PNIPAM chain collapse depends on the molecular weight and grafting density. Langmuir.

[30] Baudoin M (2019). Folding a focalized acoustical vortex on a flat holographic transducer: miniaturized selective acoustical tweezers. Sci. Adv..

[31] Lenshof A, Magnusson C, Laurell T (2012). Acoustofluidics 8: Applications of acoustophoresis in continuous flow microsystems. Lab a Chip.

[32] Rufo J, Cai F, Friend J, Wiklund M, Huang TJ (2022). Acoustofluidics for biomedical applications. Nat. Rev. Methods Prim..

[33] Schmid L, Weitz DA, Franke T (2014). Sorting drops and cells with acoustics: acoustic microfluidic fluorescence-activated cell sorter. Lab a Chip.

[34] Bruus H (2012). Acoustofluidics 7: the acoustic radiation force on small particles. Lab a Chip.

[35] Akther A, Marqus S, Rezk AR, Yeo LY (2020). Submicron particle and cell concentration in a closed chamber surface acoustic wave microcentrifuge. Anal. Chem..

[36] Collins DJ (2015). Two-dimensional single-cell patterning with one cell per well driven by surface acoustic waves. Nat. Commun..

[37] Li J (2022). Building programmable multicompartment artificial cells incorporating remotely activated protein channels using microfluidics and acoustic levitation. Nat. Commun..

[38] Collins DJ, Ma Z, Han J, Ai Y (2017). Continuous micro-vortex-based nanoparticle manipulation via focused surface acoustic waves. Lab a Chip.

[39] Ma Z (2020). Acoustic holographic cell patterning in a biocompatible hydrogel. Adv. Mater..

[40] Reboud J (2012). Shaping acoustic fields as a toolset for microfluidic manipulations in diagnostic technologies. Proc. Natl Acad. Sci. USA.

[41] Biroun MH (2019). Computational and experimental analysis of droplet transportation/jetting behaviours driven by thin film surface acoustic waves. Sens. Actuators A: Phys..

[42] Wu M (2017). Isolation of exosomes from whole blood by integrating acoustics and microfluidics. Proc. Natl Acad. Sci. USA.

[43] Wang Z (2020). Acoustofluidic salivary exosome isolation: a liquid biopsy compatible approach for human papillomavirus–associated oropharyngeal cancer detection. J. Mol. Diagnostics.

[44] Peng T (2021). Rapid enrichment of submicron particles within a spinning droplet driven by a unidirectional acoustic transducer. Anal. Chem..

[45] Akther A (2021). Acoustomicrofluidic concentration and signal enhancement of fluorescent nanodiamond sensors. Anal. Chem..

[46] Destgeer G (2016). Acoustofluidic particle manipulation inside a sessile droplet: four distinct regimes of particle concentration. Lab a Chip.

[47] Gu Y (2021). Acoustofluidic centrifuge for nanoparticle enrichment and separation. Sci. Adv..

[48] Zhang P, Bachman H, Ozcelik A, Huang TJ (2020). Acoustic microfluidics. Annu. Rev. Anal. Chem..

[49] Yang S (2022). Harmonic acoustics for dynamic and selective particle manipulation. Nat. Mater..

[50] Tian Z (2019). Wave number–spiral acoustic tweezers for dynamic and reconfigurable manipulation of particles and cells. Sci. Adv..

[51] Wang X, Qiu X, Wu C (1998). Comparison of the coil-to-globule and the globule-to-coil transitions of a single poly (N-isopropylacrylamide) homopolymer chain in water. Macromolecules.

[52] Bryzgunova OE (2016). Comparative study of extracellular vesicles from the urine of healthy individuals and prostate cancer patients. PLoS ONE.

[53] Gámez-Valero A, Lozano-Ramos SI, Bancu I, Lauzurica-Valdemoros R, Borràs FE (2015). Urinary extracellular vesicles as source of biomarkers in kidney diseases. Front. Immunol..

[54] Leiblich A (2017). Recent developments in the search for urinary biomarkers in bladder cancer. Curr. Urol. Rep..

[55] Wang S, Kojima K, Mobley JA, West AB (2019). Proteomic analysis of urinary extracellular vesicles reveal biomarkers for neurologic disease. EBioMed..

[56] Oh-Hohenhorst SJ, Lange T (2021). Role of metastasis-related microRNAs in prostate cancer progression and treatment. Cancers.

[57] Guthrie J (2008). Use of Bordetella pertussis BP3385 to establish a cutoff value for an IS 481-targeted real-time PCR assay. J. Clin. Microbiol..

[58] Sun O, Lerman LO (2020). Urinary extracellular vesicles as biomarkers of kidney disease: from diagnostics to therapeutics. Diagnostics.

[59] Simonsen JB (2017). What are we looking at? Extracellular vesicles, lipoproteins, or both?. Circulation Res..

[60] Guo F (2015). Reusable acoustic tweezers for disposable devices. Lab a Chip.

[61] Zhao S (2020). A disposable acoustofluidic chip for nano/microparticle separation using unidirectional acoustic transducers. Lab a Chip.

[62] Lighthill J (1978). Acoustic streaming. J. Sound Vib..

[63] Alghane M (2010). Experimental and numerical investigation of acoustic streaming excited by using a surface acoustic wave device on a 128° YX-LiNbO3 substrate. J. Micromech. Microeng..

