# Supplementary material for: High-yield and rapid isolation of extracellular vesicles by flocculation via orbital acoustic trapping: FLOAT
Source: Microsyst Nanoeng. 2024 Feb 4;10:23. doi: 10.1038/s41378-023-00648-3 (PMC10838941; doi:10.1038/s41378-023-00648-3)
Supplement: Supplementary file 1 — Supplementary Information [file 41378_2023_648_MOESM1_ESM.docx]

Supplementary Materials for

**High-yield and rapid isolation of extracellular vesicles by flocculation via orbital acoustic trapping: FLOAT**

Joseph Rufo, Peiran Zhang, Zeyu Wang, Yuyang Gu, Kaichun Yang, Joseph Rich, Chuyi Chen, Ruoyu Zhong, Ke Jin, Ye He, Jianping Xia, Ke Li, Jiarong Wu, Yingshi Ouyang, Yoel Sadovsky, Luke P. Lee*, & Tony Jun Huang*

*Corresponding author. Email: [lplee@bwh.harvard.edu](mailto:lplee@bwh.harvard.edu); [tony.huang@duke.edu](mailto:tony.huang@duke.edu)

**This PDF file includes the following:**

Note S1

Figs. S1 to S9

Tables S1 to S6

**Other Supplementary Materials for this manuscript include the following:**

Movies S1 to S3

**Supplementary Note S1. Modeling of nano-flocculation and orbital acoustic trapping**

As discussed in supplementary references [1] and [2], the equilibrium diameter of floc is determined by the balance between the inherent cohesive binding forces among polymer clusters and the breaking forces (hydrodynamic forces and Brownian motion), as shown in equation (1).

(1)

$$Ra= \frac{B_{f}}{{\alpha H}_{f}+\beta{Br}_{f}}$$

The threshold is estimated by equation (2).

(2)

$$Thres= \frac{{\alpha H}_{f}+\beta{Br}_{f}}{H_{f}+{Br}_{f}}$$

The cohesive binding forces are calculated with equations (3) and (4).

(3)

(4)

$$B_{f}=kd^{1+\frac{D_{f}}{3}}$$

$$k= {48}^{-\frac{2}{3}}\pi^{\frac{5}{3}}a^{\frac{2}{3}}\sigma\left( \rho_{0}-\rho_{w} \right)^{\frac{2}{3}}$$

The hydrodynamic breaking forces are calculated with equation (5).

(5)

$$H_{f}= \frac{1}{60}\pi\rho_{w}d^{4}G^{2}$$

The Brownian motion force is calculated with equation (6).

(6)

$${Br}_{f}=\zeta{(\frac{12\pi d\mu k_{B}T}{2\Delta t})}^{\frac{1}{2}}$$

The parameters used are summarized in Supplementary Table 1.


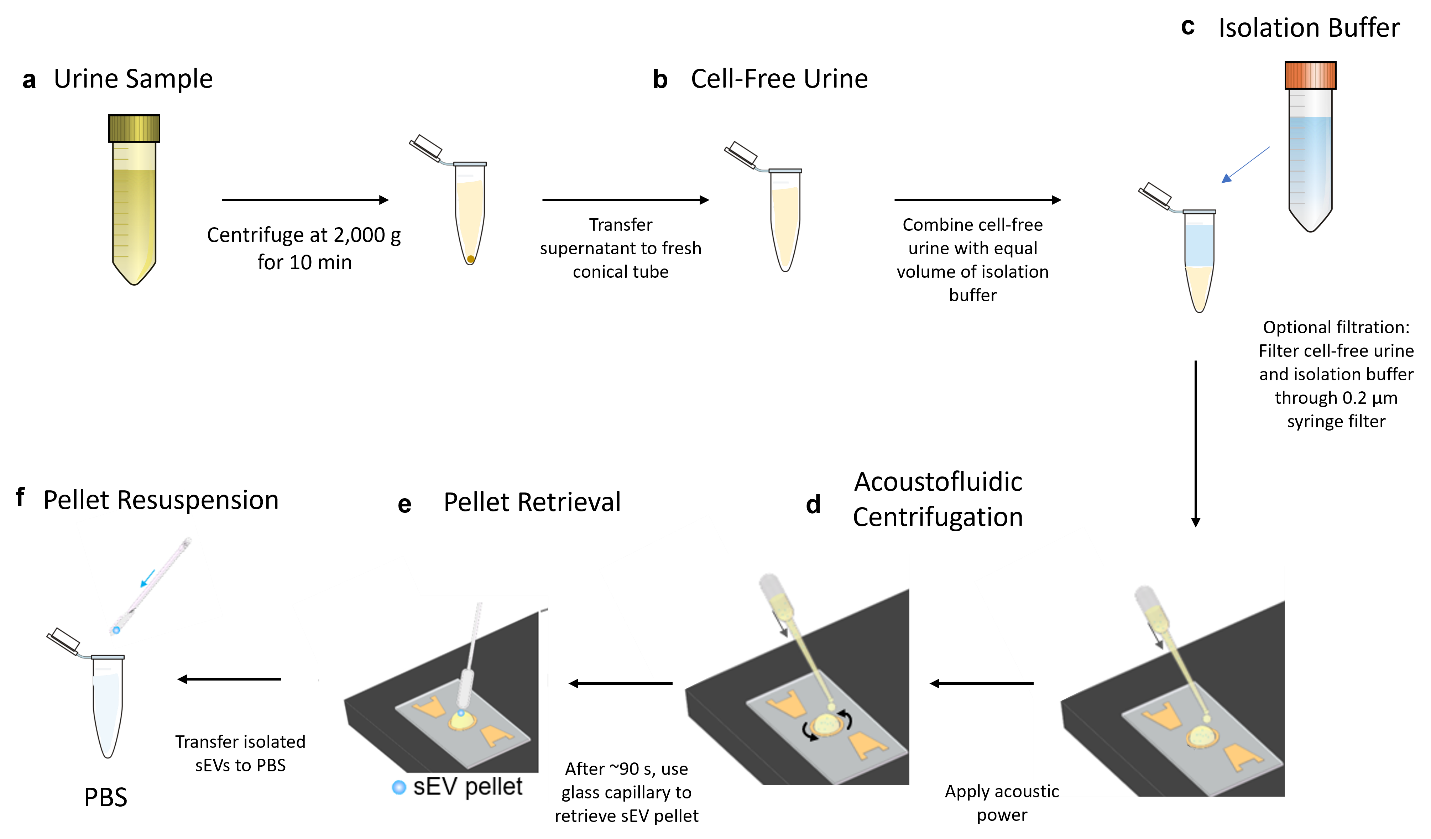


**Supplementary Figure S1:** **FLOAT procedure.** Schematic depicting the steps involved in isolating sEVs from urine using FLOAT. Starting from (a) an initial whole urine sample centrifuged to obtain (b) a cell-free urine sample. (c) Isolation buffer is added and (d) a droplet of the cell-free urine/isolation buffer solution is placed onto the acoustofluidic centrifuge. The acoustofluidic centrifuge is powered on, and the droplet begins to rotate and heat. After ~60 s, a floc of concentrated sEVs will be visible at the center of the droplet. (e) A glass capillary tube is used to retrieve the sEV pellet from the droplet and (f) resuspend the sEV pellet into PBS, where it dissociates back into individual sEVs.

**Supplementary Figure S2: Zeta potential measurements of PNIPAm and PNIPAm coated nanoparticles in various solutions.** For each solution, the zeta potential increases as the temperature increases, enabling efficient flocculation. As the zeta potential approaches zero, flocculation becomes more favorable. Error bars represent 95% confidence intervals for a sample size n=3.


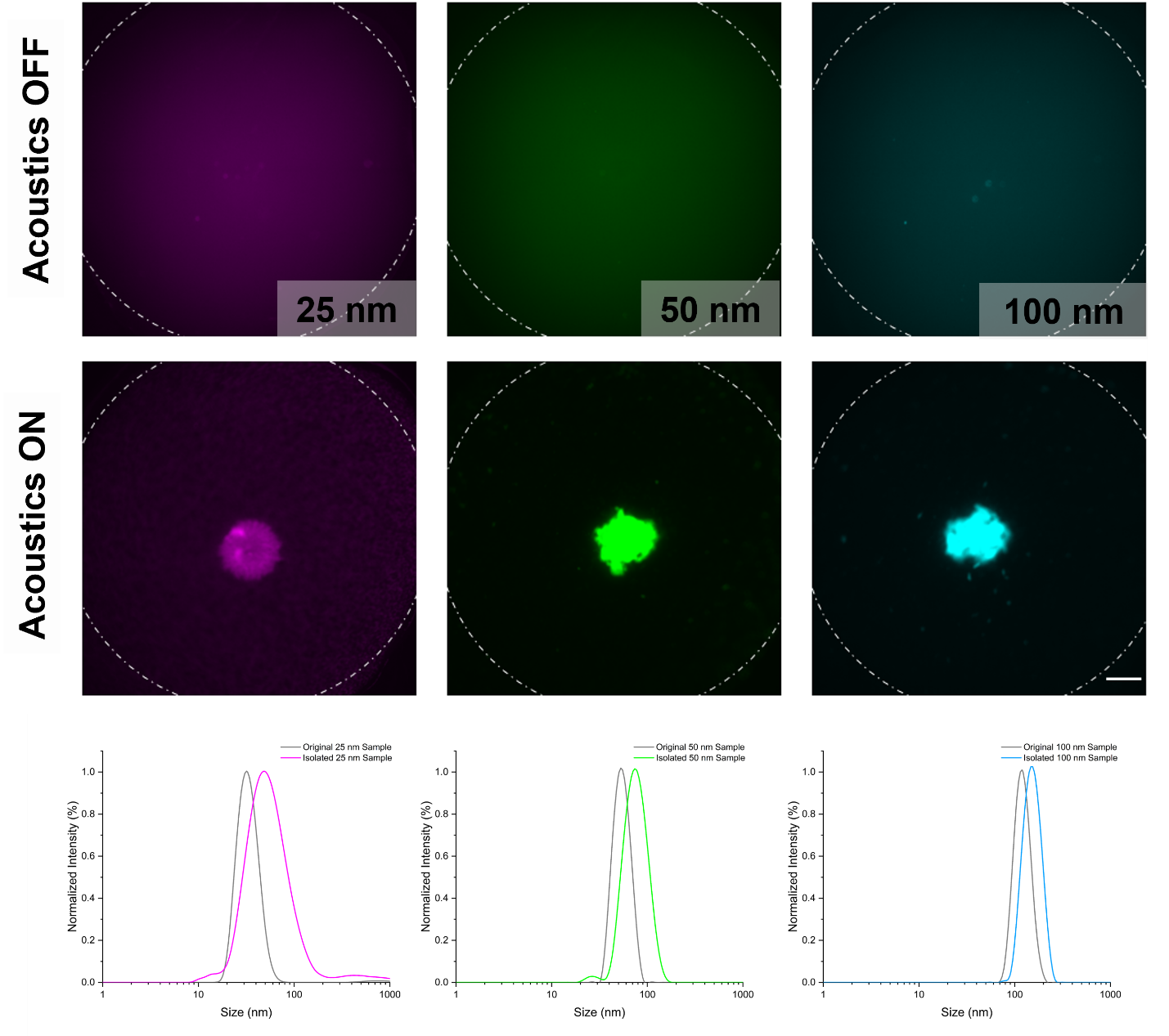


**Supplementary Figure S3:** **Concentration and Isolation of various-sized polystyrene nanoparticles.** The FLOAT platform can rapidly concentrate nanoparticles from 25 to 100 nm in diameter. Initial images are taken when the acoustics are turned off, showing the initial concentration of particles in solution. After the acoustic centrifuge is turned on and the particles are trapped at the center of the droplet, the particles are transferred to deionized water (di-H_2_O). Dynamic light scattering results are shown below each image, showing the comparison between the initial size of the nanoparticles and their size after they have been isolated and transferred, Due to the presence of the PNIPAm polymer, the average radius of the particles increases slightly. Detailed information is provided in Table S1. Scale bar: 200 µm.

**
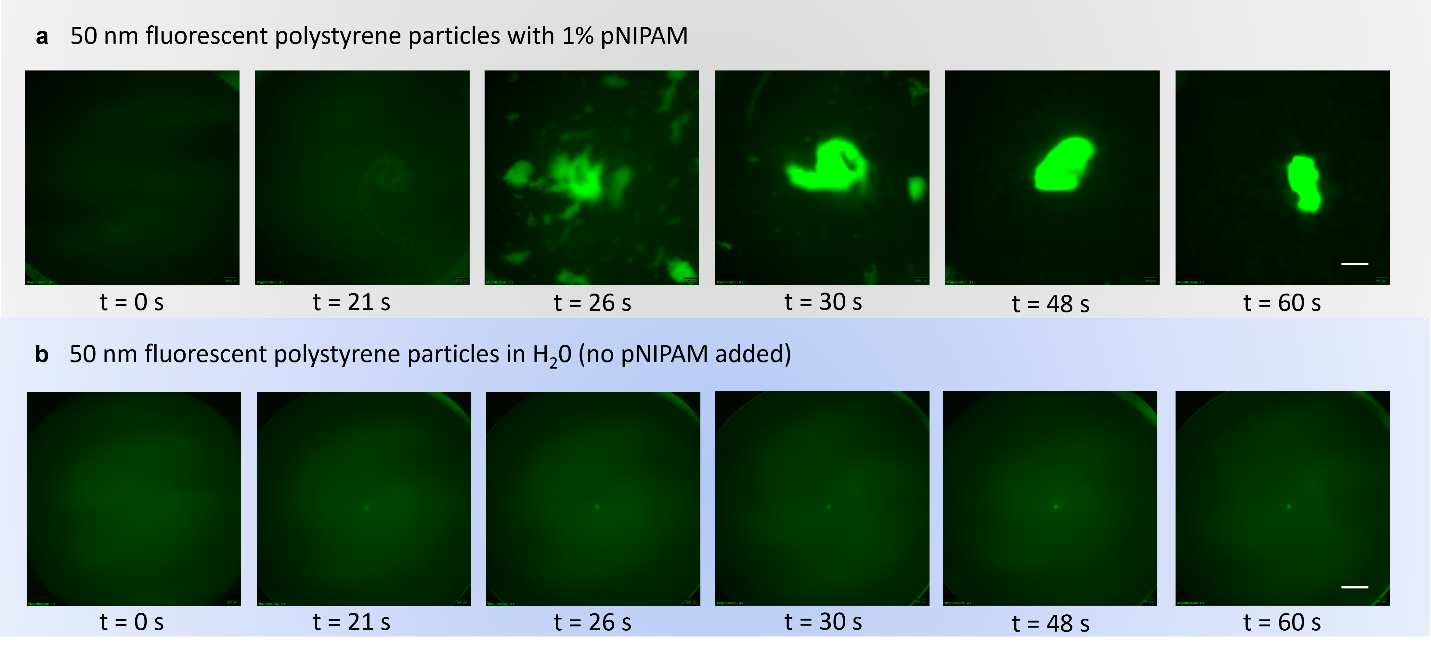
**

**Supplementary Figure S4:** **Evaluation of the concentration performance of the acoustic centrifuge in the presence and absence of PNIPAm.** To show that the concentration phenomenon observed is a result of flocculation and not acoustic concentration, we evaluated the device’s ability to concentrate 50 nm fluorescent PS particles (a) when the sample had been mixed with the PNIPAm solution before being added to the device and (b) when the sample was placed only in di-H_2_O. As seen from the time-lapsed images, when no pNIAPM is added to the solution, the particles are not concentrated at the center of the droplet. Scale bars: 200 µm.


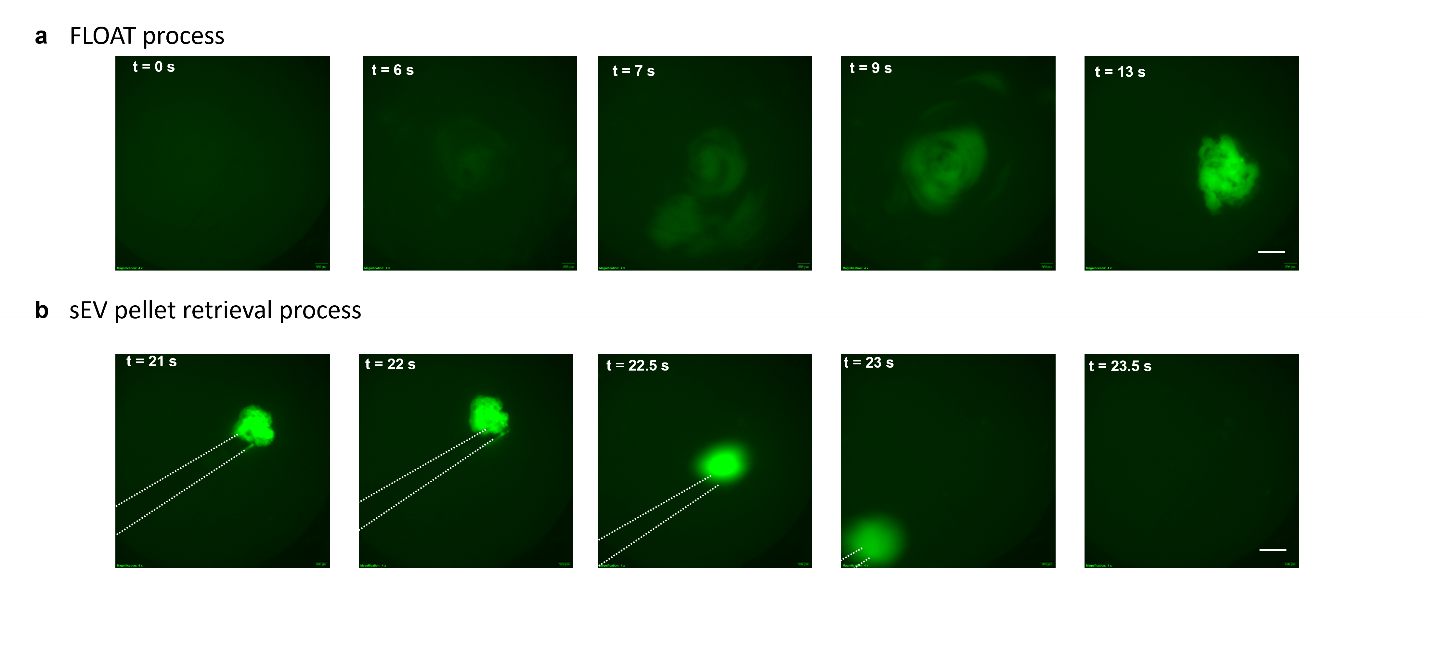


**Supplementary Figure S5:** **Time-lapse images showing the sEV pellet retrieval process.** Here, fluorescently labelled (a) sEVs are placed on the droplet centrifuge and turned on at t = 0s (Vpp = 15 V). After 13s, the sEVs can be rapidly concentrated to the center of the droplet. (b) Once the sEVs have been trapped at the center of the droplet, a glass capillary is used to manually remove the sEV pellet from the droplet and transfer it to a resuspension buffer. The pellet retrieval process takes less than 3 s to complete. Scale bars: 200 µm.


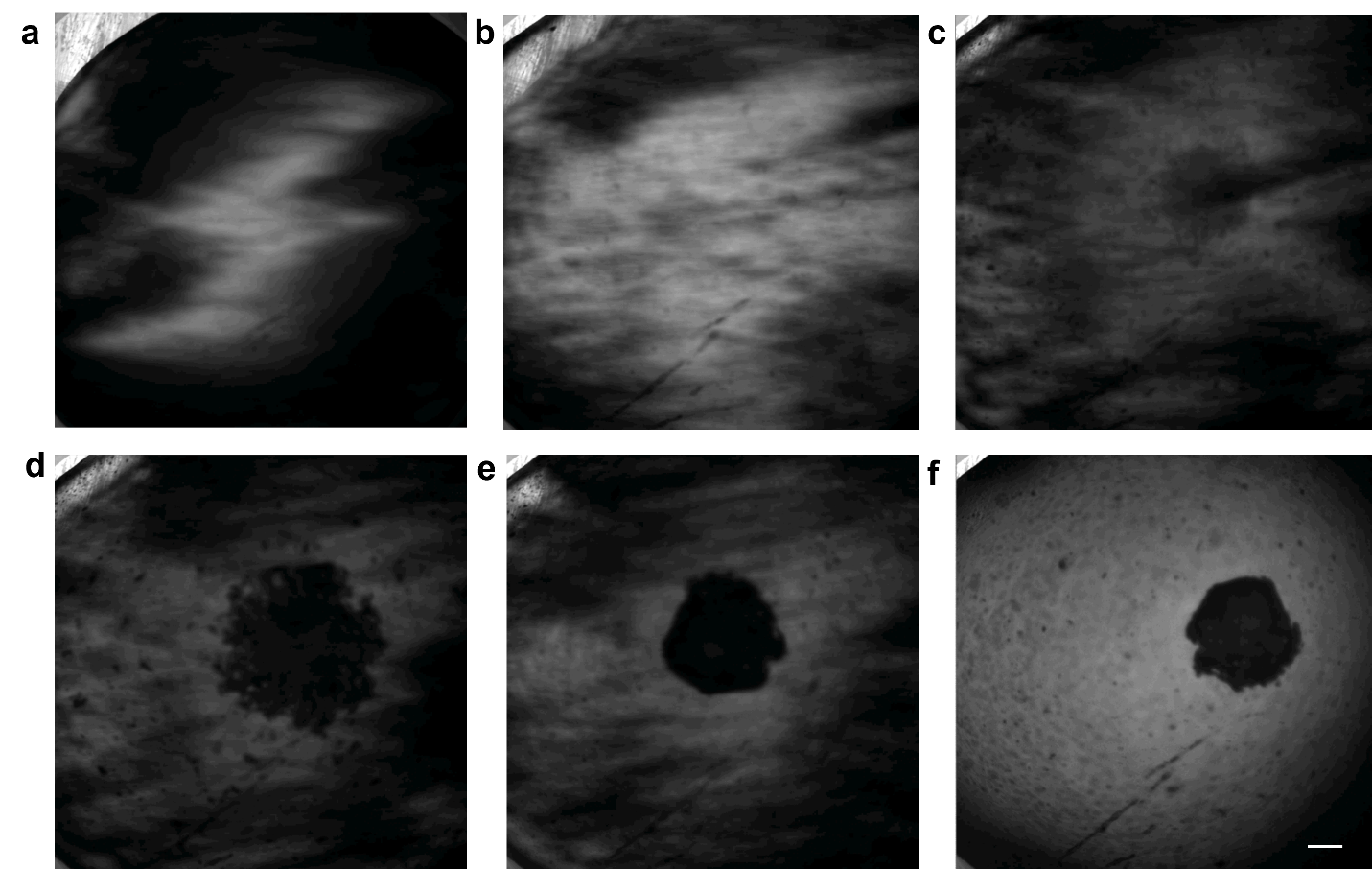


**Supplementary Figure S6:** **Time-lapse images showing the sEV pellet retrieval process from a patient urine sample.** Here, brightfield images show the FLOAT process taking place in an 8 µL urine droplet. Notable, the particle floc becomes trapped at the center of the rotating droplet and is visible to the naked eye. This allows the easy recovery and transfer of the sEV pellet using a glass capillary tube. Scale bar: 200 µm.

**
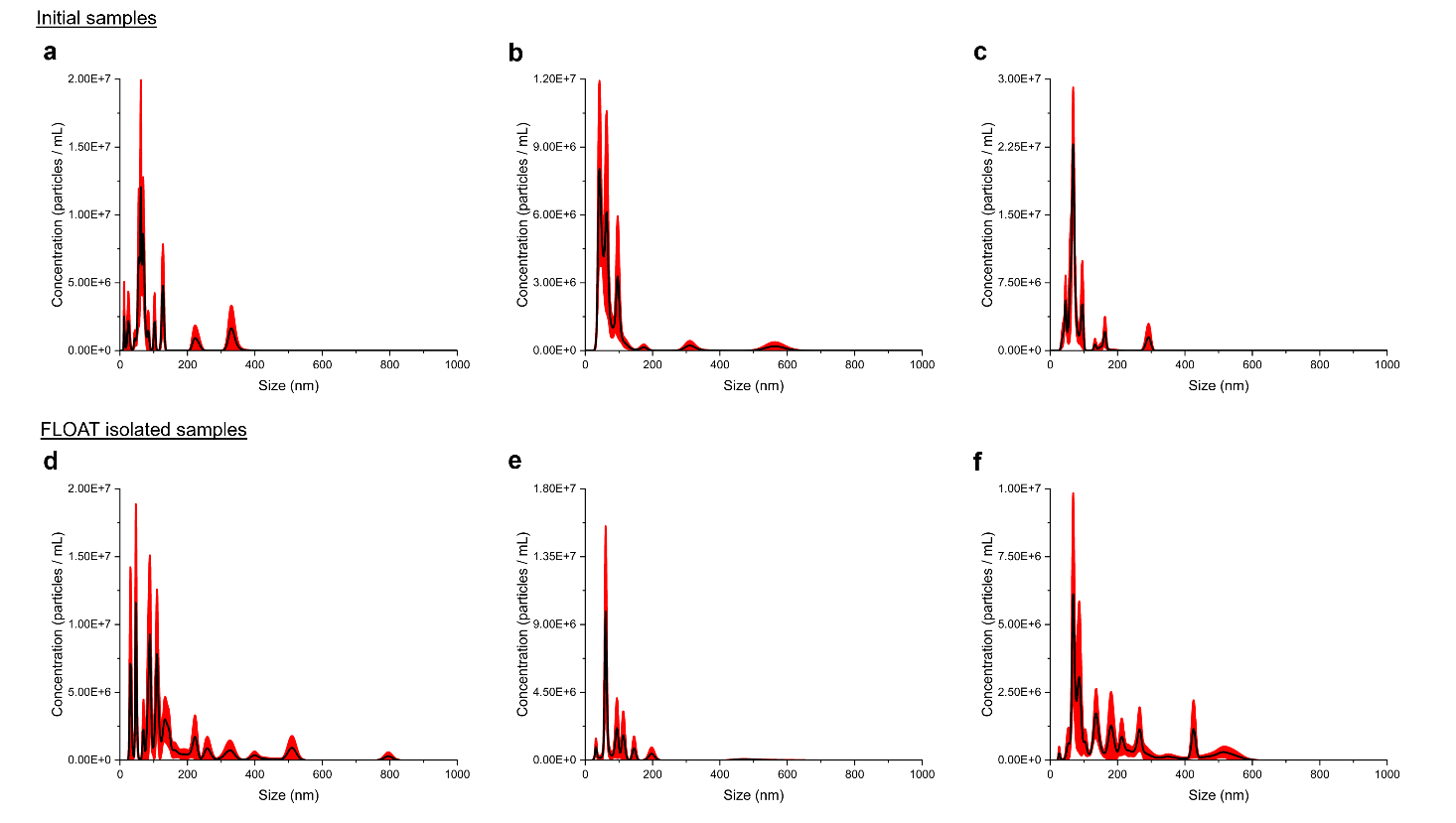
**

**Supplementary Figure S7:** **Size distribution of isolated samples.** Size distribution of (a-c) unprocessed CF urine samples and (d-f) FLOAT isolated sEV samples**.** The black line and the red area represent the fitting curve and the error bar, respectively. For each individual sample used for NTA measurement, ten subsamples are measured.


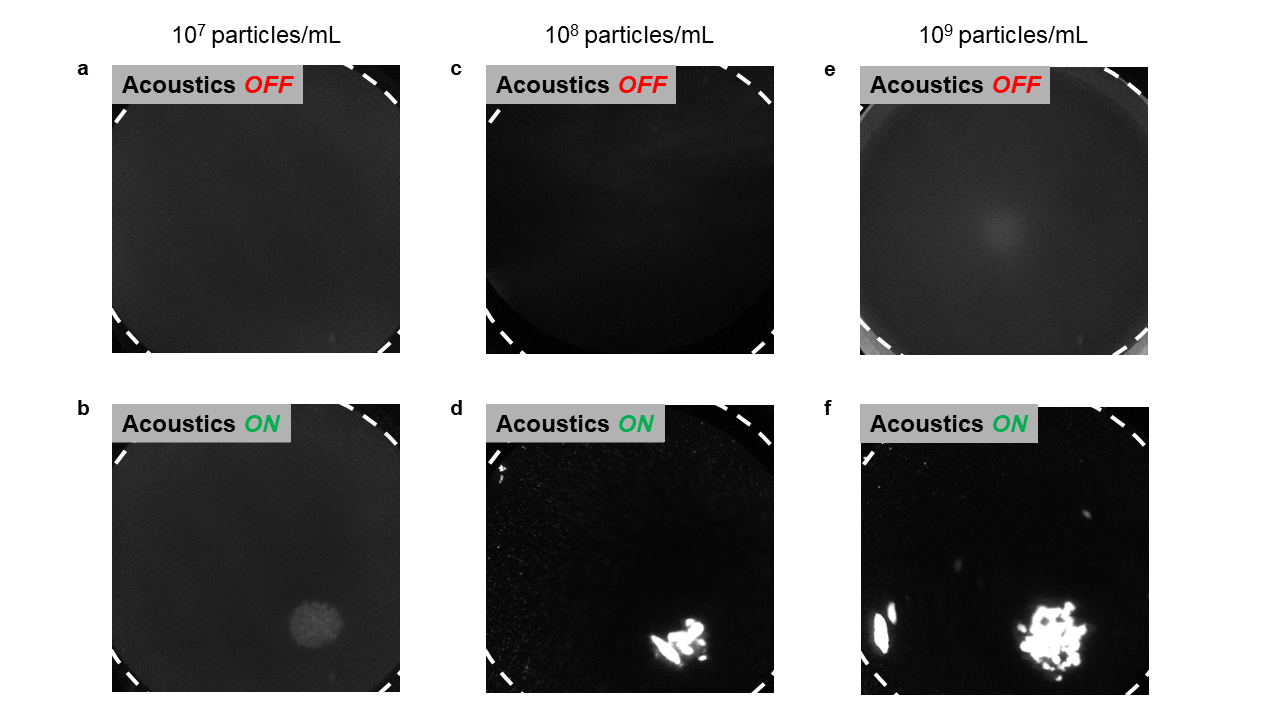


**Supplementary Figure S8:** **FLOAT performance as a function of nanoparticle concentration.** The FLOAT platform can concentrate 100 nm fluorescent polystyrene nanoparticles across 3 orders of magnitude of particle concentrations: (a-b) 10^7^, (c-d) 10^8^, and (e-f) 10^9^ particles/mL. Typical concentrations of urinary EVs in patient samples range from 10^8^ –10^9^ particles/mL. The dashed white line represents the droplet border.


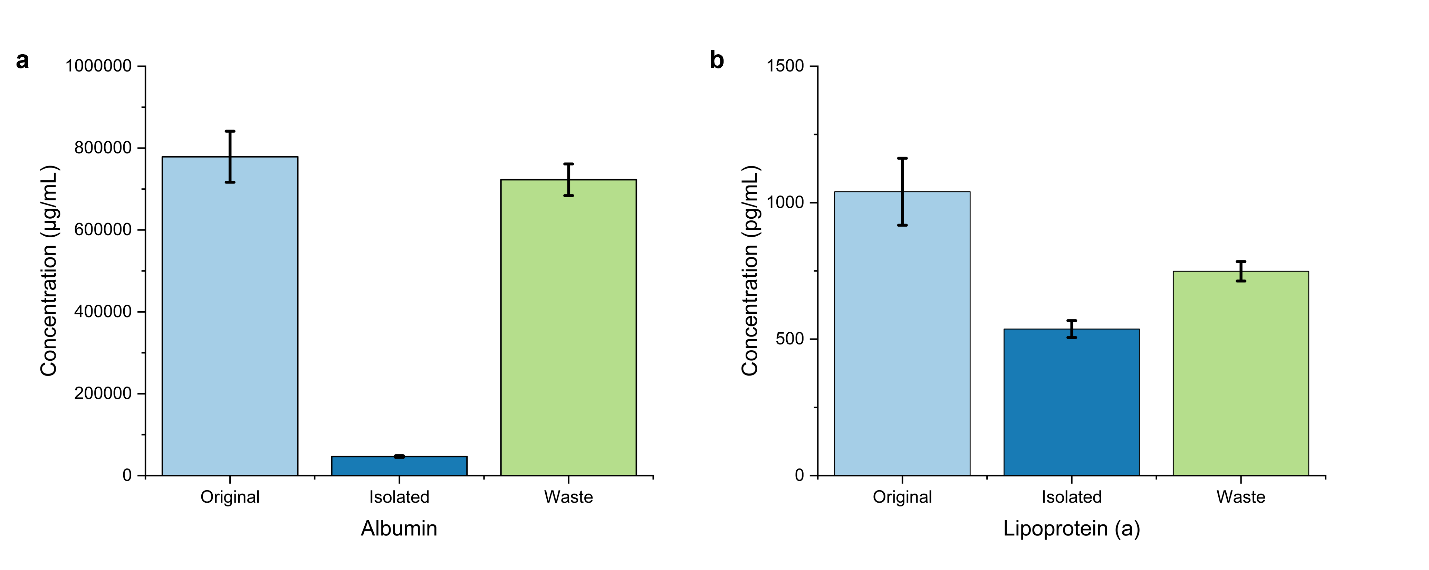


**Supplementary Figure S9.** Quantification of soluble proteins and lipoproteins in FLOAT isolated samples. Enzyme-linked immunosorbent assay (ELISA) results showing the ability of FLOAT to isolate (a) albumin and (b) lipoprotein from cell-free urine samples (noted as “original” in the figure).

| Parameter description | Parameter notation | Value | Unit |
| --- | --- | --- | --- |
| Floc diameter | *d* | 10^-2^ – 10^3^ | µm |
| Fluid volume | *V* | 10 | µL |
| Power dissipated | *P* | 5 | mW |
| Dynamic viscosity | *µ* | 1.002 | mPa⋅s |
| Average relative shearing speed | Δ*v* | 10 | mm⋅s^-1^ |
| Characteristic distance | Δ*l* | 200 | µm |
| Density function co-efficient 1 | *a* | 0.0011 | kg⋅m^-3^ |
| Density function co-efficient 2 | *κ*_p_ | 1.07 | N/A |
| Estimated particle density | *ρ*_0_ | 1,150 | kg⋅m^-3^ |
| Water density | *ρ*_w_ | 1,000 | kg⋅m^-3^ |
| Fractal dimension | *D*_f_ | 3 - 2⋅*κ*_p_ | N/A |
| Shearing coefficient | *G* | Δ*v*⋅Δ*l*^-1^ | s^-1^ |
| Global hydrodynamic stress | *σ* | *µ*⋅*G* | kg⋅m^-1^⋅s^-2^ |
| Magnitude of time-step | Δ*t* | 1 | s |
| Gaussian random number with zero mean and unit standard deviation | *ζ* | 0.7979 | N/A |
| Boltzmann constant | k_B_ | 1.38⋅10^-23^ | m^2^⋅kg⋅s^-2^⋅K^-1^ |
| Absolute temperature | *T* | 293 | K |
| Contribution coefficient for hydrodynamic shearing | *α* | 1 | N/A |
| Contribution coefficient for Brownian motion | *β* | 0.1 | N/A |

**Supplementary Table 1. Parameter values used in Fig. 2b and Note S1.**

| Sample Type | Initial particle count (kcps) | Particle count after FLOAT isolation and transfer (kcps) | Recovery Rate (%) |
| --- | --- | --- | --- |
| *25 nm polystyrene* | *221.7* | *213.8* | *96.5* |
| *50 nm polystyrene* | *143.3* | *135.1* | *95.4* |
| *100 nm polystyrene* | *215.4* | *200.5* | *93.1* |

**Supplementary Table 2: Estimation of particle recovery rate based on mean DLS particle count data.**

| Sample Type | Initial diameter (nm) | Diameter after FLOAT isolation and transfer (nm) | Thickness of shell coating (nm) |
| --- | --- | --- | --- |
| *25 nm polystyrene* | *32.63* | *49.39* | *8.38* |
| *50 nm polystyrene* | *53.14* | *70.38* | *8.62* |
| *100 nm polystyrene* | *118.13* | *143.06* | *12.46* |

**Supplementary Table 3: Change in mean particle diameter following FLOAT concentration and isolation.**

| Sample Type | Initial particle concentration (particles per mL) | Particle concentration after FLOAT isolation and transfer (particles per mL) | Recovery Rate (%) |
| --- | --- | --- | --- |
| *Fluorescently labelled urinary sEVs* | *(3.58 ± 0.64) x 10^8^* | *(3.35 ± 0.59) x 10^8^* | *93.4* |

**Supplementary Table 4: Estimation of particle recovery rate based on mean NTA particle concentration data.**

| **Parameter** | **Differential Ultracentrifugation^1,2^** | **Size Exclusion Chromatography^3^** | **Immunoaffinity^4^** | **Polymer precipitation^5^** | **Microfluidics^6,7^** | **FLOAT** |
| --- | --- | --- | --- | --- | --- | --- |
| **Mechanism** | Size, density | Size, shape | Specific binding | Solubility | Varied  Size, density, shape, charge | **Surface charge** |
| **Yield** | Moderate | High | Low | High | Moderate | **High** |
| **Purity**  **(co-isolation of soluble proteins)** | Moderate | High | High | Low | Moderate | **High** |
| **Processing Time** | 2 – 22 hours | 30 minutes | 30 min | 30 min – overnight incubation | 10 minutes – several hours | **10 minutes** |
| **Minimal Processing Volume** | 5 mL | 150 µL | 500 µL | 1 mL | 100 µL | **8 µL** |
| **Specialized equipment** | Ultracentrifuge | Chromatography column | Immunoaffinity beads/reagents | Polymer reagent | Varied  Syringe pumps, microscope | **Acoustofluidic centrifuge** |

**Supplementary Table 5:** **Comparison between methods for urinary EV isolation.**

| **Primary antibodies used for Western blotting** | | | | |
| --- | --- | --- | --- | --- |
| ***Target*** | ***Manufacturer*** | ***Host*** | ***Catalog*** | ***RRID*** |
| THP | *Santa Cruz Biotechnology* | *Mouse* | *sc-271022* | *AB_10610634* |
| CD63 | *Santa Cruz Biotechnology* | *Mouse* | *sc-5275* | *AB_627877* |
| TSG101 | *Abcam* | *Rabbit* | *ab30871* | *AB_2208084* |

**Supplementary Table 6: Primary antibodies for Western blotting.**

**REFERENCES**

1. V. Mussack, G. Wittmann, M. W. Pfaffl, Comparing small urinary extracellular vesicle purification methods with a view to RNA sequencing—Enabling robust and non-invasive biomarker research. *Biomolecular Detection and Quantification* **17**, 100089 (2019).
2. E. Tomiyama, K. Fujita, N. Nonomura, Urinary Extracellular Vesicles: Ultracentrifugation Method. *Urinary Biomarkers: Methods and Protocols*, 173-181 (2021).
3. S. Park, K. Lee, I. B. Park, N. H. Kim, S. Cho, W. J. Rhee, Y. Oh, J. Choi, S. Nam, D. H. Lee, The profiles of microRNAs from urinary extracellular vesicles (EVs) prepared by various isolation methods and their correlation with serum EV microRNAs. *diabetes research and clinical practice* **160**, 108010 (2020).
4. R. Vago, G. Radano, D. Zocco, N. Zarovni, Urine stabilization and normalization strategies favor unbiased analysis of urinary EV content. *Scientific Reports* **12**, 17663 (2022).
5. F. Royo, P. Zuñiga-Garcia, P. Sanchez-Mosquera, A. Egia, A. Perez, A. Loizaga, R. Arceo, I. Lacasa, A. Rabade, E. Arrieta, Different EV enrichment methods suitable for clinical settings yield different subpopulations of urinary extracellular vesicles from human samples. *Journal of extracellular vesicles* **5**, 29497 (2016).
6. L.-G. Liang, Y.-F. Sheng, S. Zhou, F. Inci, L. Li, U. Demirci, S. Wang, An integrated double-filtration microfluidic device for detection of extracellular vesicles from urine for bladder cancer diagnosis. *Extracellular Vesicles: Methods and Protocols*, 355-364 (2017).
7. S. Zhang, J. Deng, J. Li, F. Tian, C. Liu, L. Fang, J. Sun, Advanced microfluidic technologies for isolating extracellular vesicles. *TrAC Trends in Analytical Chemistry*, 116817 (2022).

**Supplementary Movies**

**Supplementary movie S1.** Video depicting the concentration of fluorescent 50 nm polystyrene nanoparticles within the droplet via FLOAT. Initially, the weak fluorescence signal is distributed throughout the entire droplet; after the FLOAT process, the majority of the signal is concentrated at the center of the droplet. At the end of the video, the acoustic waves are turned off, and the concentrated particles remain at the center of the droplet.

**Supplementary movie S2.** Video depicting the rotation of 50 nm particles in the absence of the PNIPAm polymer flocculant. When no flocculant is added, the particles continue to rotate and cannot be concentrated at the center of the droplet. The applied acoustic power, acoustic frequency, and liquid volume are identical to those used in supplementary video S1.

**Supplementary movie S3.** Video showing the concentration and removal of fluorescently labelled sEVs.
